# Summer Freezing Resistance: A Critical Filter for Plant Community Assemblies in Mediterranean High Mountains

**DOI:** 10.3389/fpls.2016.00194

**Published:** 2016-02-22

**Authors:** David S. Pescador, Ángela Sierra-Almeida, Pablo J. Torres, Adrián Escudero

**Affiliations:** ^1^Departamento de Biología y Geología, Física y Química Inorgánica, Universidad Rey Juan CarlosMóstoles, Spain; ^2^ECOBIOSIS, Departamento de Botánica, Facultad de Ciencias Naturales y Oceanográficas, Universidad de ConcepciónConcepción, Chile; ^3^Instituto de Ecología y BiodiversidadSantiago, Chile

**Keywords:** abiotic filter, alpine plants, drought, freezing events, functional diversity, functional traits, leaf dry matter content, LT_50_

## Abstract

Assessing freezing community response and whether freezing resistance is related to other functional traits is essential for understanding alpine community assemblages, particularly in Mediterranean environments where plants are exposed to freezing temperatures and summer droughts. Thus, we characterized the leaf freezing resistance of 42 plant species in 38 plots at Sierra de Guadarrama (Spain) by measuring their ice nucleation temperature, freezing point (FP), and low-temperature damage (LT_50_), as well as determining their freezing resistance mechanisms (i.e., tolerance or avoidance). The community response to freezing was estimated for each plot as community weighted means (CWMs) and functional diversity (FD), and we assessed their relative importance with altitude. We established the relationships between freezing resistance, growth forms, and four key plant functional traits (i.e., plant height, specific leaf area, leaf dry matter content (LDMC), and seed mass). There was a wide range of freezing resistance responses and more than in other alpine habitats. At the community level, the CWMs of FP and LT_50_ responded negatively to altitude, whereas the FD of both traits increased with altitude. The proportion of freezing-tolerant species also increased with altitude. The ranges of FP and LT_50_ varied among growth forms, and only leaf dry matter content was negatively correlated with freezing-resistance traits. Summer freezing events represent important abiotic filters for assemblies of Mediterranean high mountain communities, as suggested by the CWMs. However, a concomitant summer drought constraint may also explain the high freezing resistance of species that thrive in these areas and the lower FD of freezing resistance traits at lower altitudes. Leaves with high dry matter contents may maintain turgor at lower water potential and enhance drought tolerance in parallel to freezing resistance. This adaptation to drought seems to be a general prerequisite for plants found in xeric mountains.

## Introduction

Freezing temperature events during the growing season are not rare in alpine habitats (Billings, 1974), so survival at such temperatures could be considered a primary abiotic determinant in these areas (Körner, 2003). Several studies have demonstrated that alpine plant species have the ability to resist freezing events during the summer growing season at temperatures ranging from –4°C to –10°C in temperate and high latitude mountains (Sakai and Otsuka, 1970; Taschler and Neuner, 2004; Körner and Alsos, 2008), while some plant species in mountains with dry summers can even survive at temperatures close to –20°C (Squeo et al., 1996; Sierra-Almeida et al., 2009; Venn et al., 2013). The physiological ability to cope with these events must also be remarkable in Mediterranean type mountains because for instance the minimum temperatures at the treeline in central Spain (i.e., 1900 m at Navacerrada Pass) can often drop to –8°C in May (Supplementary Table S1), thereby suggesting that freezing events may occasionally drop to –11°C at the summits (above 2400 m), especially during the early growing season. Thus, summer freezing events may be considered critical for the assembly of plant communities in these alpine-type habitats. Indeed, environmental filtering implies a certain level of plant functional trait convergence because abiotic conditions limit some critical functional traits to certain values (Pavoine et al., 2011). If this is the case, then the functional diversity (FD) of traits related to freezing resistance (e.g., leaf freezing point and lethal temperature) will decline as the temperatures decreases (e.g., higher altitudes). Furthermore, the community weighted means (CWMs hereafter), which reflect the average value at the community level for a specific plant trait considering the abundance per species (Violle et al., 2007; Lepš et al., 2011), are expected to decrease with altitude for freezing resistance traits simply because the frequency of freezing events will increase with it, and thus species with high freezing resistance (Nagy and Grabherr, 2009). Accordingly, a “macroclimatic hypothesis" has been proposed to explain the pattern of freezing resistance mechanisms (i.e., tolerant versus avoidant species; Sakai and Larcher, 1987) with altitude. In general, freezing-tolerant species should become dominant with altitude, where freezing events are longer, more intense, and frequent during the growing season, whereas avoidant species would be confined to lower altitudes (Squeo et al., 1996; Sierra-Almeida et al., 2009). In addition, the freezing risk limitation on plant survival may be explained by a “microclimate hypothesis" which suggests that short plant growth forms are exposed to lower, longer, and more frequent freezing events near the ground than tall growth forms (Larcher, 2003). This vertical thermal gradient means that short plants (e.g., rosettes and cushion plants) are more likely to be tolerant with a high freezing resistant (Squeo et al., 1991, 1996), whereas tall plants (e.g., giant rosettes and shrubs) should be avoidant with low freezing resistance (Rada et al., 1985b; Squeo et al., 1991, 1996).

Assessments of the altitudinal patterns and plant differences in freezing resistance are particularly interesting in Mediterranean-type mountains because plant survival is restricted by a double climatic limitation in these habitats: sporadic freezing events and generalized summer drought (Cavieres et al., 2006; Giménez-Benavides et al., 2007). Given that soil water shortages are more pronounced at lower altitudes in these mountains and that freezing and drought responses may be physiologically correlated (Beck et al., 2007), the expected patterns in the FD and CWMs of traits related to freezing resistance with altitude may shift from the expectations of the macroclimate hypothesis or they could simply be neutralized. Furthermore, although a correlation with some morphological traits (i.e., plant height) was previously postulated for the so-called microclimatic hypothesis, adaptations to the stressful conditions that are typical of these mountains (i.e., drought and freezing events) should also depend on specific coordinated or trade-off relationships with others plant traits (Reich et al., 2003; Violle et al., 2007). Consequently, correlations between freezing resistance traits and other key plant functional traits such as the leaf-height-seed combination can be critical for understanding the functional response to freezing events during the growth activity period. In particular, a negative correlation between plant height and freezing resistance was reported in plants from the tropical high Andes (Squeo et al., 1991). It has also been proposed that plant species with a low specific leaf area (SLA) and a high leaf dry matter content (LDMC) will have a reduced water loss area (Lopez-Iglesias et al., 2014), more structural material, and increased resistance to physical stress (Cornelissen et al., 2003). This may result in greater cell wall negative turgor pressures, and thus an increase in freezing resistance (Anisko and Lindstrom, 1996). Furthermore, it has been reported that cold acclimation has led to decreases in the SLA of some Mediterranean legume species, thereby increasing their freezing resistance (Hekneby et al., 2006). Moreover, the seed mass is positively correlated with the competitive ability and resistance to abiotic stress (Verhoeven et al., 2004). Thus, plant species that produce larger seeds should be more freezing resistant than those that produce smaller seeds.

Our hypothesis is that unpredictable freezing events during the summer growing season are a major assembly filter in most high mountains, but that this is not necessarily the case in Mediterranean climate type mountain communities. This could be a consequence of the critical role played by the summer drought, which may be an important additional filter that acts in the opposite direction on these communities, particularly because the physiological responses of plants to both events are related. Thus, we predict that the FD and CWMs of traits linked to freezing resistance will not decrease with altitude, and that the traits associated with drought tolerance will be positively correlated with freezing resistance. Therefore, we studied the summer freezing resistance of members of a Mediterranean high mountain community by determining their freezing resistance (i.e., magnitude and mechanisms), the potential role of this ability in the community assembly (especially in terms of CWMs and FD responses), and their correlations with other key plant functional traits. In particular, we tested: (i) whether summer freezing events represent an abiotic filter that affects the community FD and CWMs and the extent to which their responses along altitude are biased by the typical Mediterranean summer drought; (ii) whether freezing-tolerant species are more abundant than avoidant species at higher altitudes, as suggested by the macroclimatic hypothesis; (iii) whether freezing resistance differences exist among growth forms (i.e., short and tall species resist different freezing temperatures), as predicted by the microclimatic hypothesis; and (iv) whether freezing resistance traits are correlated with other plant functional traits, i.e., height, LDMC, SLA, and seed mass, most of which are related to water economy.

## Materials and Methods

### Study Area and Target Species

The study was performed in cryophilic grassland communities in the Sierra de Guadarrama National Park, which is approximately 70 km northwest of Madrid, Spain. The climate is Mediterranean with an average annual temperature and precipitation of 6.4°C and 1350 mm, respectively (Supplementary Table S1; Navacerrada Pass weather station; 40° 47′ N, 4° 0′ W; 1894 m a.s.l.). A regular and extreme summer drought occurs from July to August (i.e., <10% of the total annual rainfall), which is particularly intense at lower altitudes (Giménez-Benavides et al., 2007).

During the summer of 2011, we sampled 38 cryophilic grassland communities dominated by *Festuca curvifolia* Lag. *ex* Lange. These communities were located in Guadarrama National Park above a timberline formed by stunted Scots pines (*Pinus sylvestris* L.) between 1900 and 2000 m, which was interspersed with a shrubby matrix formed by *Cytisus oromediterraneus* Rivas Mart. & al. and *Juniperus communis* L. subsp. *alpina* (Suter) Celak. The sampled communities were distributed along the whole mountain range and they covered the entire altitudinal gradient (1940-2428 m a.s.l.), different orientations, slopes, and pasture and shrub cover types. These communities were dominated by several creeping chamaephytes and graminoids, which were structured in a biphasic mosaic of plants that formed conspicuous patches or stripes on a bare ground matrix. In each grassland, we established a plot of 20 m × 20 m, where we measured the cover of each plant species (we distinguished between shrub cover and pasture cover) and the percentage of bare soil. We estimated the altitude using a GPS (Garmin Colorado-300).

In 2012, we collected plant samples from four of the richest plots sampled in the previous year. Two were located at the edges (i.e., the highest summit, Peñalara at 2419 m a.s.l, and the area around Navacerrada Pass at 1860 m) and the other two were at intermediate altitudes, i.e., the Bola del Mundo summit at 2242 m and Hermana Mayor peak at 2280 m. In total, 42 plant species belonging to 17 families were collected from these plots (Supplementary Table S2). These species represented the most abundant species in the cryophilic grasslands surveyed and they encompassed a wide range of phylogenetic and functional types. In particular, we sampled about 65% of the species present in this community, which covered >90% of the relative abundance. The plant species corresponded to four different growth forms: cushions (8), graminoids (8), forbs (23), and shrubs (3).

### Freezing Resistance Analyses

Plant material was collected between July 11 and 27, during the peak of the growing season 2012. Freezing resistance analyses were performed using six green fully expanded leaves per individual, thereby ensuring that the analyses employed mature, healthy, and active leaves. The leaves were collected from five mature individuals per species, which were selected randomly in the field (see Sierra-Almeida et al., 2010 for more details of the sample collection criteria). In the case of clonal species, the leaves of each individual were selected from the same rootball belonging to an independent and conspicuous vegetated patch. Immediately after collection, the plant samples were covered with wet paper in sealed and tagged plastic bags, and then placed in a cooler to prevent changes in the tissue water status, before being transported to the laboratory at Rey Juan Carlos University. The samples were kept cold in a domestic refrigerator (i.e., 4°C) until the freezing resistance analyses.

#### Thermal Analyses

One leaf from each individual (i.e., five leaves per species) was used to determine the ice nucleation temperature (NT) and freezing point (FP). Each leaf was bound to a thermocouple (Gage 30 copper-constantan thermocouples; Cole Palmer Instruments, Vernon Hills, IL, USA) with parafilm and placed in a closed cryotube. The cryotubes were immersed into a cryostat (F34-ME, Julabo Labortechnik GmbH, Seelbach, Germany), which was programmed to decrease the temperature of the antifreeze solution (Husky, Würth, Germany) from 0 to –19°C at a cooling rate of 2°C per hour. The temperatures of individual leaves were recorded live each second with a Personal Daq/56 multi-channel thermocouple USB data acquisition module (IOtech, Cleveland, OH, USA), which was equipped with a PDQ2 expansion module and connected to a laptop. The sudden rise in leaf temperature (exotherm) produced by the heat released during the extracellular freezing process was used to determine the NT and FP. The first temperature corresponds to the lowest temperature before the exotherm, which indicates the onset of ice crystal formation. The FP represents the highest point of the exotherm, which indicates the freezing of water in the apoplast, including symplastic water driven outward by the water potential difference caused by apoplastic ice formation (Larcher, 2003).

#### Freezing Temperature Damage

For each species, five leaves detached from different plant samples were used at each target temperature to estimate the freezing temperature damage (LT_50_). Each leaf was placed in a small marked resealable plastic bag. Each small bag containing a single leaf belonging to the same species was placed in a bigger plastic bag. This procedure was used to separate and identify the different individuals from each species. It was necessary to exclude the air from the bags and to add some ballast to avoid flotation. The leaves were incubated in a previously cooled cryostat (F34-ME, Julabo Labortechnik GmbH, Germany). The cryostat was set at four different target temperatures: –5, –10, –15, and –19°C. For each target temperature, all of the leaves were transferred from the refrigerator to the cryostat and incubated for 2 h to reach a homogeneous leaf temperature. After the freezing treatment, the plastic bags were removed from the cryostat and placed in the refrigerator in darkness at 4°C for 24 h to thaw. As a control, we used leaves that were kept in darkness and stored in plastic bags at 4°C for 24 h without the freezing treatment. This method of direct rather than gradual cooling and thawing (i.e., from 4°C to the target temperatures, and *vice versa*) measures the current freezing resistance rather than the hardening capacity of the plant material (Larcher et al., 2010), and thus it may have similar effects on plant leaves as the freezing events that occur in the field, where we have observed cooling rates higher than 4-6°C⋅h^–1^, particularly at the beginning and end of the growing season. Visual damage was not immediately obvious in all species, so leaf damage was assessed after thawing by using a chlorophyll fluorimeter (Fluorescence Monitoring System FMS 2, Hansatech, Germany) to obtain the ratio of variable relative to maximum fluorescence (*F_v_*/*F_m_*) for a dark-adapted leaf. A dead leaf effectively had an *F_v_*/*F_m_* of zero (Bannister et al., 2005), so plant damage was calculated as the percentage of photoinactivation (100 ×*PhI*). The photoinactivation ratio was defined by Larcher (2000) as: *PhI* = (1-*F_fT_*/*F_max_*), where *F_fT_* is the *F_v_*/*F_m_* obtained for the sample exposed to freezing temperature T and *F_max_* is the maximum *F_v_*/*F_m_* obtained for the individuals, including the control. LT_50_ corresponds to the temperature at which *PhI* reaches a 50% value in leaf samples, which we determined by linear interpolation using the temperature of the highest *PhI* < 50% and the temperature of the lowest *PhI* > 50% (Bannister et al., 1995; Bannister et al., 2005). This method has been used in non-invasive surveys of the effects of thermal stress on photosynthesis and the results obtained agreed with those produced by other methods when assessing the LT_50_ (e.g., visual score and vital stain) in alpine plants (Neuner and Buchner, 1999).

#### Freezing Resistance Mechanism

The NT and LT_50_ values for each species were compared to estimate the physiological freezing resistance mechanism. When LT_50_ was significantly lower than NT, the species was considered to be freezing tolerant (FT). When LT_50_ was not significantly different from NT, the species was considered to be freezing avoidant (FA) (Sakai and Larcher, 1987). In some plant species, LT_50_ occurred before NT and these species were considered to be freezing sensitive (FS).

### Measurements of Other Plant Functional Traits

We measured four additional functional traits for most of the species studied (38 species; see Supplementary Table S3 for more details) and to test their correlations with freezing resistance traits. We measured traits that represent key components of plant fitness and that reflect plant ecological strategies, i.e., plant height (Hmax, distance between the ground and the topmost photosynthetic tissues), SLA (ratio of the leaf fresh area relative to its dry leaf mass), LDMC (ratio of the leaf dry mass relative to its fresh mass), and seed mass. Plant materials were sampled from 10 randomly selected mature and healthy individuals during the growing seasons 2011 and 2012 from the same four plots at Sierra de Guadarrama, according to standardized protocols (Cornelissen et al., 2003). For each individual, we weighed two fresh well-developed leaves using a microbalance (Mettler Toledo MX5, Columbus, OH, USA; weight uncertainty ± 1 μg). The projected surface area of each fresh leaf was estimated using a digital scanner (Epson Perfection 4870, Seiko Epson Corp., Nagano, Japan) and Adobe Photoshop CS3 software (Adobe Systems, San Jose, CA, USA). Next, the leaves were oven-dried at 60°C for 72 h and the dry mass was measured. Finally, 30 additional individuals per species were selected randomly at Sierra de Guadarrama and their seeds were collected. In total, 30 seeds from each species were dried in resealable plastic bags containing silica gel, closed hermetically, and then stored at environmental temperature for 1 month before measuring their mass using a microbalance.

### Plant Community Data

In order to evaluate the roles of freezing resistance traits in the assembly of Mediterranean alpine plant communities along environmental gradients, we employed information collected from 38 cryophilic grassland plots. Using the FP and LT_50_ values and the species cover from each plot, we estimated the CWMs for these plant traits (Violle et al., 2007; Lepš et al., 2011) and the FD for each plot and freezing trait. The CWMs represent the mean trait value in a community when considering the relative abundance of each species at each site (weighted by species cover) and can be estimated as:

$$CWM=\overset{s}{\underset{i=1}{\Sigma }}p_{i}x_{i}$$

where S is the number of species in a given plot, *p_i_* is the cover of the i-th species in each plot and *x_i_* is the trait value of the i-th species. We also calculated the FD for each separate trait based on Rao’s quadratic entropy index (Rao index; Rao, 1982), where for a specific trait space, this index represents the sum of pairwise distances among all possible pairs of species present in a community weighted by the species cover according to:

$${FD}_{Rao}=\;\overset{s}{\underset{i=1}{\Sigma }}\overset{s}{\underset{j=1}{\Sigma }}p_{i}p_{j}d_{ij}$$

where *d_ij_* expresses the trait dissimilarity between each pair of coexisting species *i* and *j*. The distances among species were standardized between 0 when the species were functionally equivalent and one when the species differentiation was maximized. So, we used a Gower distance matrix, as suggested by Pavoine et al. (2009). We estimated the CWMs and Rao components without considering the *F. curvifolia* cover. This species is a genuine ecosystem engineer that occurred in all sites at very high cover rates and it could obscure the functional roles of the other species.

### Statistical Analyses

To determine differences in the freezing resistance mechanisms based on NT and LT_50_, we performed *t*-tests and Mann-Whitney *U*-tests when the necessary assumptions were not met. The proportions of FT and FA species were determined in each plot and their changes with altitude were evaluated using linear regression models. The response of each functional component (CWMs and FD) along altitude were tested using linear models. Differences in freezing resistance traits (i.e., FP and LT_50_) among different growth forms were compared using Kruskal-Wallis rank-sum tests and honestly significant differences between pairwise growth forms were evaluated by a *post hoc* Nemenyi test. Finally, the relationships among freezing resistance and other plant functional traits were evaluated using Pearson’s or Spearman’s correlations.

All of the statistical analysis were conducted using R 2.12.1 (R Development Core Team, 2010). The one-way test function in the coin package (Hothorn et al., 2008) was employed for Nemenyi test calculations. The dbFD function implemented in the FD package (Laliberté et al., 2014) was used to calculate the CWMs and FD values.

## Results

The freezing resistance of 42 species from Mediterranean alpine habitats encompassed a broad range of responses (Supplementary Table S2). NT values varied widely within and between the growth forms and ranged from –13.3 ± 0.5°C in the shrub *Adenocarpus complicatus* to –4.7 ± 0.3°C in the graminoid *Nardus stricta*. LT_50_ varied from –18.6 ± 0.2°C in the graminoid *Deschampsia flexuosa* to –5.1 ± 0.1°C in the forb *Coincya monensis* subsp. *cheiranthos*. FP also exhibited high variation, ranging from –6.9 ± 0.6°C in the graminoid *Koeleria crassipes* to –0.6 ± 0.2°C in the forb *Biscutella valentina* subsp. *Pyrenaica* (Supplementary Table S2). Moreover, FP was positively correlated with LT_50_ (Spearman’s *R* = 0.42, *P* = 0.007).

**FIGURE 1:**
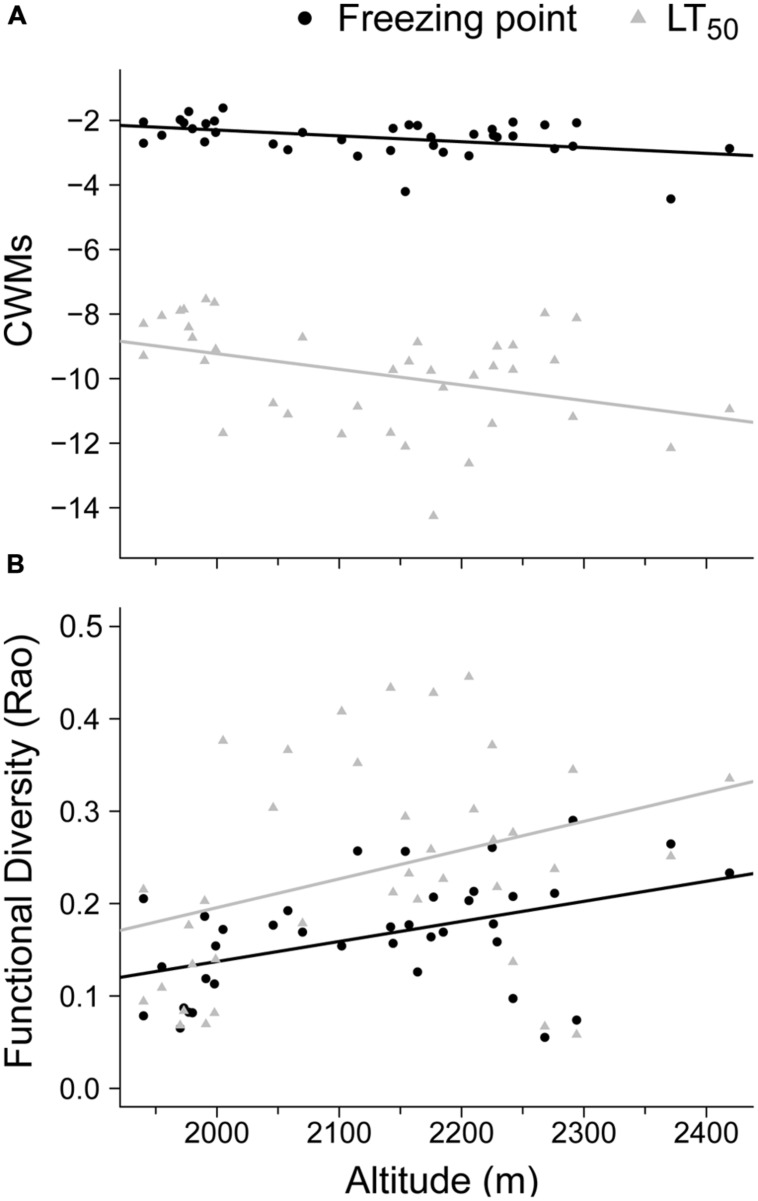
**Community response to summer freezing resistance based on different functional components and the altitudinal gradient considered across 38 plots.** The significant relationships between altitude and: **(A)** community weighted means (CWMs) and **(B)** functional diversity (FD – Rao) are shown for the freezing point (FP, °C; solid circles) and freezing temperature damage (LT_50_, °C; gray triangles). Lines represent the significant regression linear model slopes.

The community survey showed that the two functional components (CWMs and FD) of the freezing resistance traits (FP and LT_50_) were significantly related to altitude (**Figure 1**). In particular, the models for the CWMs of FP and LT_50_ indicated a significant negative relationship with altitude (*R^2^* = 0.17, *P* = 0.01 and *R^2^* = 0.15, *P* = 0.02, respectively; **Figure 1A**). The FD metric (i.e., Rao) had a positive relationship with altitude for both FP and LT_50_ (*R^2^* = 0.20, *P* = 0.004, and *R^2^* = 0.12, *P* = 0.032, respectively; **Figure 1B**).

Almost half of the plant species (47.6%) were classified as FT and 42.9% were FA. Only a tiny fraction of plants were sensitive to freezing damage (FS - 9.5%). The FT proportion increased with altitude (*R^2^* = 0.22, *P* = 0.002), ranging from 30% at low altitude to 60% at the highest plots while the FA proportion exhibited the opposed pattern since the number of FS species was almost negligible.

**FIGURE 2:**
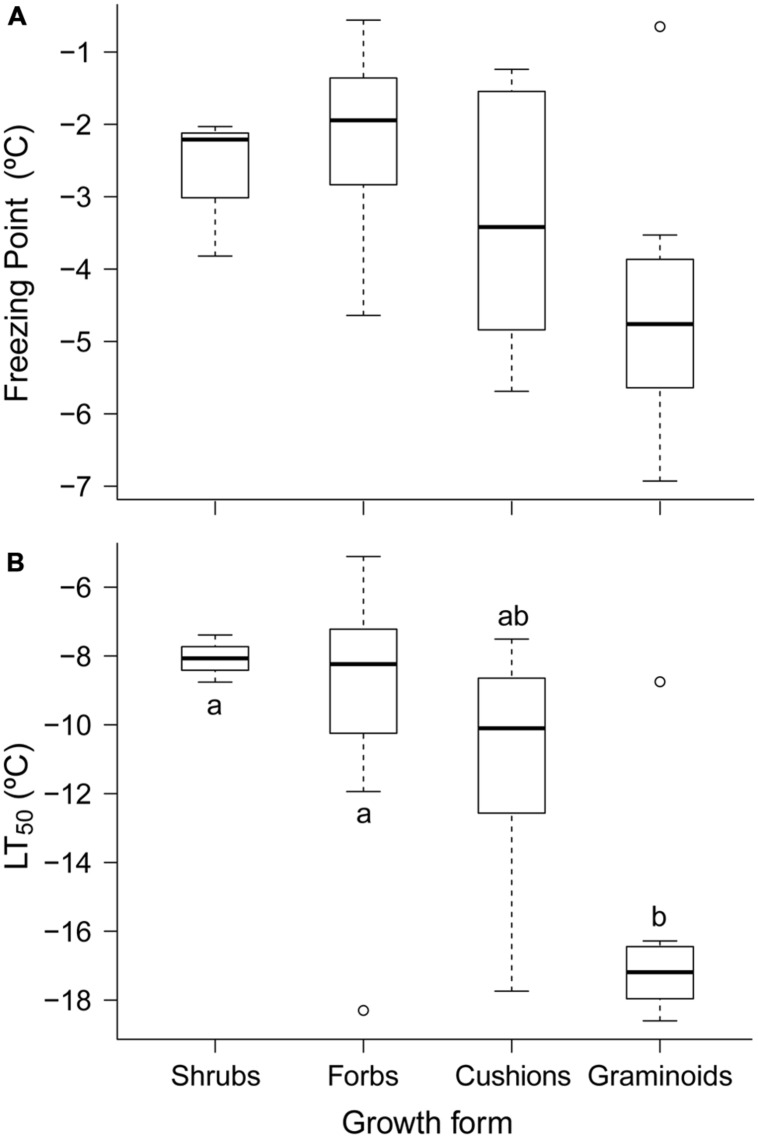
**Summer freezing resistance measured in the leaves of 42 species and grouped by growth form: **(A)** freezing point (FP, °C) and **(B)** freezing temperature damage (LT_50_, °C).** Each box plot shows the median (solid line), interquartile range (box enclosure), variability outside the upper and lower quartiles, which is no more than 1.5 times the length of the box away from the box (extreme “*whisker”*), and outliers (black points). Pairs of box plots labeled with different letters above the “*whisker*” differ significantly (*P* < 0.05). Significant differences were evaluated using a *post hoc* Nemenyi test within each growth form.

The ranges of FP and LT_50_ varied among growth forms (**Figure 2**). Graminoids species comprised the group with the most freezing resistance, where the average LT_50_ was 7.2 K and 6.3 K lower than that for shrubs and forbs, respectively. Cushions were ranked in an intermediate position, where the average LT_50_ was 4.3 K higher than that for graminoids (**Figure 2B**). Furthermore, the FPs of our species exhibited a similar LT_50_ pattern, although only graminoids and forbs were significantly different (**Figure 2A**). However, plant height alone did not explain the differences between growth forms because the Spearman’s correlation coefficients between plant height and FP or LT_50_ were not significant.

**FIGURE 3:**
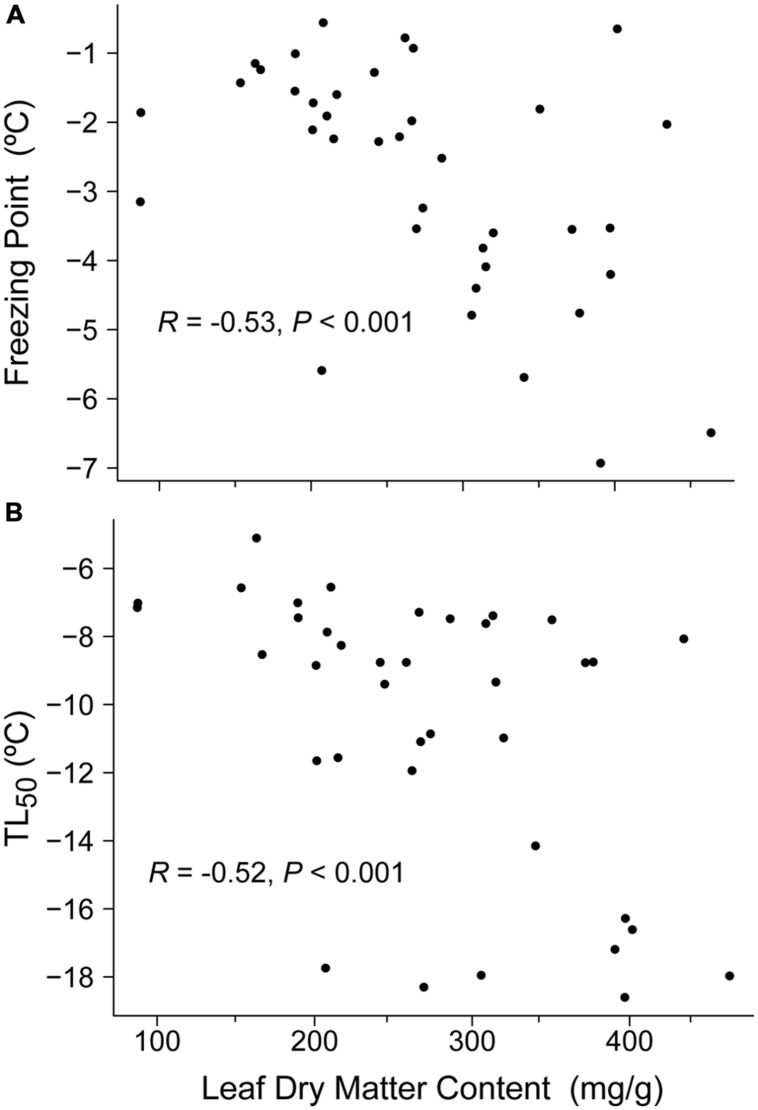
**Relationships between leaf dry matter content (LDMC, mg/g) and: **(A)** freezing point (FP, °C) and **(B)** freezing temperature damage (LT_50_, °C).** Results of Spearman’s correlation are provided.

Leaf dry matter content was the only functional plant trait that had a significant correlation with freezing resistance traits. Hence, LDMC was negatively correlated with FP and LT_50_ (Spearman’s *R* = –0.52, *P* < 0.001 and Spearman’s *R* = –0.53, *P* < 0.001, respectively; **Figure 3**).

## Discussion

Freezing resistance is critical for plant life in alpine habitats and an important determinant of the structure and functioning of these communities. Although, very few multi-species studies have investigated the ability for freezing resistance (but see Taschler and Neuner, 2004; Bannister et al., 2005; Sierra-Almeida et al., 2009, 2010; Venn et al., 2013), our results demonstrated the importance of summer freezing events for the organization and assembly of Mediterranean high mountain communities, where we found that freezing resistance traits and their functional components (i.e., CWMs and FD) appeared to be structured with altitude. However, the characteristic concomitant and pronounced drought at lower altitudes in Mediterranean-type mountains probably interfered with this response.

Mediterranean high mountain species had a broad LT_50_ range (Supplementary Table S2) where more than 60% of species could resist temperatures below –8°C, which is the absolute minimum air temperature recorded during the growing season in the study area over the last 40 years at the lowest limit of the community (1840 m in the Navacerrada Pass; Supplementary Table S1). This implies that the majority of species can cope with freezing temperatures during the growing season that are much lower than the temperatures reached at present. The average LT_50_ of these species (-8.8°C) is similar to that reported for other European alpine species (i.e., Austrian Alps species average LT_50_ = –8.1°C; Taschler and Neuner, 2004), but their wide LT_50_ range and their extreme values (i.e., <-18°C) in particular are closer to those reported from mountains with dry summers (Sierra-Almeida et al., 2009; Venn et al., 2013). Thus, these Mediterranean species are among the most freezing-resistant plants described to date. These extraordinarily high levels of resistance are probably related to the integration of traits required to survive extreme water shortages during the summer period (soil water content <5% at 1900 m, Giménez-Benavides et al., 2007).

At the community level, the freezing resistance values weighted by species abundance decreased with altitude (**Figure 1A**), thereby suggesting a response to a summer coldness filter. The entrance of more freezing-resistant plants (e.g., *Agrostis rupestris*, *Phyteuma hemisphaericum*, or *Veronica fruticans* subsp. *cantabrica*) and the increased cover by *Minuartia recurva* or *Silene ciliata* at higher altitudes explain this decrease. The response of the FD of freezing resistance traits along gradients was particularly interesting (**Figure 1B**). The decrease in FD for FP and LT_50_ at low altitudes suggests the local coexistence of species with similar freezing trait values and a reduced range for these freezing traits. This finding may be unexpected given that an increase in stress with altitude is considered to be the norm in alpine habitats (Körner, 2007), and thus the FD should have decreased as stress increased (Weiher and Keddy, 1995; Cornwell and Ackerly, 2009; but see Spasojevic and Suding, 2012). Two non-exclusive explanations are possible. First, the mountain range investigated in the present study is not very high (only 2419 m) and the summit could not constitute a real freezing edge, so this may explain why the expected drop with altitude was not found (Taschler and Neuner, 2004; Sierra-Almeida et al., 2009). Second, the typical summer drought in Mediterranean mountains increases as the altitude decreases (Giménez-Benavides et al., 2007), and this constraint may be more relevant for plant life than the increase in coldness with altitude (Cavieres et al., 2006; Schöb et al., 2013). A close correlation between various freezing attributes and other features related to drought tolerance would help to explain this outcome.

The freezing resistance mechanisms were distributed evenly among the species studied, where the proportion of FT species increased with altitude while proportion of FA species decreased, which is consistent with the macroclimatic hypothesis. Interestingly, the FT mechanism is also predominant among species in the Chilean Andes, especially at higher altitudes where freezing events are more severe and of longer duration (Squeo et al., 1996; Sierra-Almeida et al., 2009, 2010). A first explanation comes from the altitudinal temperature decrease in alpine habitats (Nagy and Grabherr, 2009), which exposes plants to harsh thermal conditions (Sierra-Almeida et al., 2009), but a second explanation arises from the drought which also varies with altitude. Given that this is an operational mechanism classification, the higher proportion of FA plants at low altitudes may be related mainly to a decrease in NT, which depends on the tissue water status and especially on their structure (e.g., size and cell layers localization; Larcher, 2003; Wisniewski et al., 2014). Therefore, the higher intensity and frequency of droughts at low altitudes may affect the plant water status (passive osmotic adjustment), where there is less water to freeze in the tissues, thereby selecting for freezing avoidance (Sierra-Almeida et al., 2009). However, values of NT were very variable within and between growth forms and biogeographic origin, suggesting that this trait may be more related with phylogenetic constraints at the tissue structure of species inhabiting low altitudes.

Freezing resistance also appeared to be related to the growth strategy, where the highest and lowest FP and LT_50_ values varied from forbs to graminoids, respectively. A similar pattern was found in the Austrian Alps (Taschler and Neuner, 2004), where graminoids were also the most FT species (see also Körner, 2003; Bannister, 2005), but the freezing resistance of our graminoids was higher than that in other alpine habitats (Taschler and Neuner, 2004; Sierra-Almeida et al., 2009; Venn et al., 2013). This is probably attributable to their high leaf toughness and sclerophyllous habit, which are known functional traits related to drought resistance that are typical of Mediterranean plants (Gullo and Salleo, 1988). In addition, Squeo et al. (1991) reported a negative correlation between plant height and freezing-resistance in Puna-type Andean plants. However, our results agree with those obtained in other Mediterranean areas (Sierra-Almeida et al., 2010), where there were no correlations with plant height, rejecting the microclimatic hypothesis. Thus, a relationship between plant height and freezing resistance did not appear to be present in our community in this dry ecosystem, where the extreme summer droughts probably favor xeromorphic traits over plant height (Sierra-Almeida et al., 2010).

As mentioned above, the ecological performance under stressful conditions is determined by a combination of responses by several traits (MacGillivray et al., 1995; Violle et al., 2007). Thus, a correlation is expected between freezing resistance and other functional traits due to phenotypic integration. For example, LDMC had a significant and negative correlation with FP and LT_50_. This trend suggests that a convergence between morpho-anatomic and physiological traits is triggered by drought. Several studies have reported that the presence of water-soluble carbohydrates can depress the FP (Rada et al., 1985a; Alberdi et al., 1989) and that their accumulation is positively related to drought tolerance. In addition, plants exposed to drought or/and freezing may synthesize and accumulate solutes (Streeter et al., 2001; Monson et al., 2006), and/or thicken and waterproof their cell walls, thereby increasing the LDMC values and decreasing the water content in the intercellular spaces. Thus, leaves with a high dry matter content, which are often thicker, rigid, and with a higher structural material content (Cornelissen et al., 2003), can maintain turgor at a lower leaf water potential (Cheung et al., 1975; Monson and Smith, 1982) to induce greater water stress tolerance (Engelbrecht and Kursar, 2003) as well as freezing resistance (resistance to freeze dehydration; Anisko and Lindstrom, 1996). Thus, this result reinforces the idea that drought and freezing resistance are closely related.

## Conclusion

To the best of our knowledge, this is the first study to show that responses to freezing summer events are shown as critical for the assembly of alpine plant communities. The linear response of CWMs for freezing traits with altitude and the increase in FT species with altitude support the macroclimate hypothesis. In summary, the ability to resist freezing temperatures is crucial for passing this primary abiotic filter. However, our findings also suggest that the regular summer droughts that characterize Mediterranean systems may explain the high levels of freezing resistance observed in these habitats as well as the decrease in FD for FP and LT_50_ at low altitudes. Overall, these findings reinforce the hypothesis that freezing and drought resistance are linked, and that both condition the plant community assemblies in Mediterranean-type mountains. Our results are also relevant in a climate change context because increases in mean temperatures may threaten the plant species found in alpine habitats directly and indirectly. For example, decreases in freezing resistance due to warmer air temperatures (Sierra-Almeida and Cavieres, 2010) and increases in plant vulnerability to freezing damage because of early snowmelt (Martin et al., 2010; Gerdol et al., 2013) may lead to strong impacts of climate change on the structure of plant communities at high altitudes. Moreover, if we consider an opposite altitudinal gradient for water shortages in Mediterranean alpine habitats, then predicting the community and species responses to climate change will be even more complex. The correlation between LDMC and freezing resistance traits suggests that drought could ameliorate or override the negative effects of warming in this respect. However, this might only be true provided that the warmer temperatures are not accompanied by an increase in summer precipitation in Mediterranean alpine habitats.

## Author Contributions

DP conceived and designed the study, conducted the data collection, analyzed the data, and edited the manuscript; AS contributed to the study conception, designed, and performed the experiments and edited the manuscript; PT conducted the data collection, performed the experiments, contributed to the data analysis, and manuscript approval; AE contributed to the study conception, the data analysis, and manuscript approval.

## Conflict of Interest Statement

The authors declare that the research was conducted in the absence of any commercial or financial relationships that could be construed as a potential conflict of interest.
