# Supplementary material for: Summer Freezing Resistance: A Critical Filter for Plant Community Assemblies in Mediterranean High Mountains
Source: Front Plant Sci. 2016 Feb 22;7:194. doi: 10.3389/fpls.2016.00194 (PMC4761790; doi:10.3389/fpls.2016.00194)
Supplement: Supplementary file 1 [file Table_1.DOCX]

Table S1. Climate information for the study region from 1946 to 2011 (Navacerrada Pass weather station; 40°47′35′′N, 4°0′38′′W; 1896 m a.s.l.). Source: *Agencia Estatal de Meteorología - Ministerio de Agricultura, Alimentación y Medio Ambiente* of Spain.

|  | **Annual** | **Growing season** | **May** | **June** | **July** | **August** | **Sep** |
| --- | --- | --- | --- | --- | --- | --- | --- |
| Mean air temperature (°C) | 6.48 | 12.88 | 7.05 | 12.29 | 16.42 | 16.20 | 12.43 |
| Mean minimum air temperature (°C) | 2.77 | 8.18 | 3.09 | 7.65 | 11.15 | 10.96 | 8.06 |
| Absolute minimum air temperature (°C) | -20.30 | -8.00 | -8.00 | -3.40 | 0.00 | 0.20 | -3.00 |
| Mean maximum air temperature (°C) | 10.18 | 17.56 | 11.00 | 16.90 | 21.67 | 21.42 | 16.79 |
| Absolute maximum air temperature (°C) | 31.80 | 31.80 | 25.40 | 29.00 | 30.80 | 31.80 | 30.80 |
| Precipitation (mm/month) | 109.86 | 64.61 | 128.52 | 69.11 | 24.19 | 27.55 | 71.33 |
| Mean days of rain | 67.45 | 33.17 | 8.34 | 8.24 | 4.42 | 5.20 | 8.40 |
| Mean days of snow | 72 | 6 | 5 | 1 | 0 | 0 | 0 |
| Mean days of hail | 5 | 7 | 3 | 2 | 1 | 1 | 1 |
| Insolation (hours/day) | 6.07 | 8.99 | 6.97 | 9.21 | 11.16 | 10.40 | 7.13 |
| Insolation (%) | 48.09 | 63.52 | 48.02 | 61.26 | 75.58 | 75.41 | 57.00 |
| Days/month with air T < 0°C | 12 | 2 | 8 | 1 | 0 | 0 | 1 |
