# Supplementary material for: Summer Freezing Resistance: A Critical Filter for Plant Community Assemblies in Mediterranean High Mountains
Source: Front Plant Sci. 2016 Feb 22;7:194. doi: 10.3389/fpls.2016.00194 (PMC4761790; doi:10.3389/fpls.2016.00194)
Supplement: Supplementary file 2 [file Table_2.DOCX]

Table S2. Freezing resistance in the leaves of 42 species from Sierra de Guadarrama (Spain) during the growing season. Parameters: NT, ice nucleation temperature; FP, freezing point; LT_50_, temperature producing 50% damage. Values are shown as mean ± SE. Mechanisms (Mech.): FA, freezing avoidance; FT, freezing tolerance; FS, freezing sensitive. Growth form abbreviations: Sh., shrub; Forb; Cs., cushion; and Gram., graminoid.

| Species | Family | Growth form | NT (°C) | FP (°C) | LT_50_ (°C) | Mech. |
| --- | --- | --- | --- | --- | --- | --- |
| *Adenocarpus complicatus* (L.) J. Gay in Durieu | Fabaceae | Sh. | –13.32 ± 0.5 | –3.82 ± 0.17 | –7.39 ± 0.08 | FS |
| *Agrostis delicatula* Pourr. ex Lapeyr. | Poaceae | Gram. | –8.54 ± 0.27 | –4.2 ± 0.58 | –16.28 ± 0.45 | FT |
| *Agrostis rupestris* All. | Poaceae | Gram. | –8.18 ± 0.4 | –4.76 ± 0.5 | –8.75 ± 0.08 | FA |
| *Alchemilla saxatilis* Buser | Rosaceae | Forb | –8.58 ± 0.42 | –3.55 ± 0.61 | –8.77 ± 0.38 | FA |
| *Armeria caespitosa* (Gómez Ortega) Boiss. in DC. | Plumbaginaceae | Cs. | –6.66 ± 0.35 | –1.81 ± 0.16 | –7.51 ± 0.3 | FA |
| *Biscutella valentina* subsp. *pyrenaica* (A. Huet) Grau & Klingenberg | Brassicaceae | Forb | –5.37 ± 0.48 | –0.56 ± 0.18 | –7.87 ± 0.33 | FT |
| *Campanula herminii* Hoffmanns. & Link | Campanulaceae | Forb | –8.67 ± 0.45 | –2.24 ± 0.62 | –11.56 ± 0.8 | FT |
| *Coincya monensis* subsp. *cheiranthos* (Vill.) Aedo, Leadlay & Muñoz Garm. in Castrov. & al. (eds.) | Brassicaceae | Forb | –7.98 ± 0.4 | –1.15 ± 0.29 | –5.11 ± 0.08 | FS |
| *Cytisus oromediterraneus* Rivas Mart. & al. | Fabaceae | Sh. | –8.89 ± 0.77 | –2.21 ± 0.71 | –8.76 ± 0.56 | FA |
| *Deschampsia flexuosa* (L.) Trin. | Poaceae | Gram. | –7.76 ± 0.25 | –3.53 ± 0.2 | –18.6 ± 0.23 | FT |
| *Dianthus lusitanicus* Brot. | Caryophyllaceae | Cs. | –7.94 ± 0.16 | –3.24 ± 0.3 | –10.86 ± 0.85 | FT |
| *Digitalis purpurea* subsp *purpurea* L. | Scrophulariaceae | Forb | –6.07 ± 0.09 | –1.29 ± 0.12 | –8.21 ± 0.25 | FT |
| *Erysimum penyalarense* (Pau) Polatschek. | Brassicaceae | Forb | –7.79 ± 0.79 | –1.72 ± 0.3 | –11.65 ± 0.17 | FT |
| *Festuca curvifolia* Lag. ex Lange | Poaceae | Gram. | –10.1 ± 0.67 | –6.49 ± 0.74 | –17.97 ± 0.43 | FT |
| *Gentiana lutea* L. | Gentianaceae | Forb | –7.6 ± 0.49 | –0.93 ± 0.44 | –11.09 ± 0.32 | FT |
| *Herniaria glabra* L. | Illecebraceae | Forb | –8.08 ± 0.31 | –4.18 ± 0.16 | –8.95 ± 0.31 | FA |
| *Jasione crispa* (Pourr.) Samp. | Campanulaceae | Cs. | –6.06 ± 0.22 | –1.28 ± 0.15 | –8.76 ± 0.41 | FT |
| *Juniperus communis* subsp. *alpina* (Suter) Célak | Cupressaceae | Sh. | –7.27 ± 0.5 | –2.03 ± 0.31 | –8.07 ± 0.07 | FA |
| *Jurinea humilis* (Desf.) DC. | Asteraceae | Forb | –6.68 ± 0.41 | –1.98 ± 0.37 | –7.29 ± 0.78 | FA |
| *Koeleria crassipes* Lange | Poaceae | Gram. | –11.27 ± 0.69 | –6.93 ± 0.63 | –17.19 ± 0.61 | FT |
| *Leucanthemopsis alpina* (L.) Heywood | Asteraceae | Forb | –8.46 ± 0.54 | –1.6 ± 0.25 | –8.26 ± 0.25 | FA |
| *Linaria saxatilis* (L.) Chaz. | Scrophulariaceae | Forb | –7.91 ± 0.51 | –1.91 ± 0.2 | –6.55 ± 0.96 | FA |
| *Luzula hispanica* Chrtek & Krísa | Juncaceae | Gram. | –9.4 ± 0.53 | –4.79 ± 0.42 | –17.95 ± 0.37 | FT |
| *Minuartia recurva* (All.) Schinz & Thell. | Caryophyllaceae | Cs. | –10.25 ± 0.11 | –5.69 ± 0.3 | –14.15 ± 0.34 | FT |
| *Murbeckiella boryi* (Boiss.) Rothm. | Brassicaceae | Forb | –6.91 ± 0.33 | –1.43 ± 0.25 | –6.57 ± 0.58 | FA |
| *Nardus stricta* L. | Poaceae | Gram. | –4.68 ± 0.33 | –0.65 ± 0.29 | –16.61 ± 0.58 | FT |
| *Paronychia polygonifolia* (Vill.) DC. in Lam. et DC. | Illecebraceae | Cs. | –8.58 ± 0.39 | –4.09 ± 0.52 | –9.34 ± 0.28 | FA |
| *Phyteuma hemisphaericum* L. | Campanulaceae | Forb | –10.79 ± 0.51 | –3.54 ± 0.68 | –18.3 ± 0.3 | FT |
| *Pilosella castellana* (Boiss. & Reut.) F.W. Schultz & Sch. Bip. | Asteraceae | Forb | –6.91 ± 0.68 | –0.78 ± 0.22 | –11.94 ± 0.74 | FT |
| *Pilosella vahlii* (Froel.) F.W. Schultz & Sch. Bip. | Asteraceae | Forb | –8.51 ± 0.21 | –2.11 ± 0.39 | –8.85 ± 0.07 | FA |
| *Rumex acetosella* L. | Polygonaceae | Forb | –8.51 ± 0.64 | –1.55 ± 0.14 | –7.01 ± 0.5 | FA |
| *Saxifraga pentadactylis* subsp. *willkommiana* (Boiss. ex Willk.) Rivas Mart. | Saxifragaceae | Cs. | –9.3 ± 0.74 | –1.24 ± 0.35 | –8.53 ± 0.3 | FA |
| *Sedum brevifolium* DC. | Crassulaceae | Forb | –10.63 ± 0.67 | –3.15 ± 0.41 | –7.15 ± 0.55 | FS |
| *Sedum candollei* Raym.-Hamet | Crassulaceae | Forb | –8.59 ± 0.35 | –1.86 ± 0.47 | –7.02 ± 0.51 | FS |
| *Senecio boissieri* DC. | Asteraceae | Forb | –11.03 ± 0.42 | –4.64 ± 0.7 | –11.14 ± 1.14 | FA |
| *Senecio carpetanus Boiss & Reuter* | Asteraceae | Forb | –8.62 ± 0.66 | –1.01 ± 0.31 | –7.45 ± 0.14 | FA |
| *Silene boryi* Boiss. | Caryophyllaceae | Forb | –8.43 ± 0.39 | –2.28 ± 0.14 | –9.4 ± 0.69 | FA |
| Table S2. Cont. |  |  |  |  |  |  |
|  |  |  |  |  |  |  |
| *Silene ciliata* Pourr. | Caryophyllaceae | Cs. | –11.93 ± 0.38 | –5.59 ± 0.36 | –17.74 ± 0.51 | FT |
| *Solidago virgaurea* subsp. *fallit–tirones* (Font Quer) Rivas Mart. | Asteraceae | Forb | –7.93 ± 0.62 | –2.52 ± 0.39 | –7.48 ± 0.63 | FA |
| *Taraxacum* sp. | Asteraceae | Forb | –7.16 ± 0.41 | –2.13 ± 0.3 | –9.31 ± 0.3 | FT |
| *Thymus praecox* subsp. *penyalarensis (Pau) Rivas Mart.* | Lamiaceae | Cs. | –8.39 ± 0.41 | –3.6 ± 0.45 | –10.98 ± 0.94 | FT |
| *Veronica fruticans* subsp. *cantabrica* M. Laínz | Scrophulariaceae | Forb | –9.49 ± 0.3 | –4.4 ± 0.14 | –7.62 ± 0.03 | FT |
