# Supplementary material for: Summer Freezing Resistance: A Critical Filter for Plant Community Assemblies in Mediterranean High Mountains
Source: Front Plant Sci. 2016 Feb 22;7:194. doi: 10.3389/fpls.2016.00194 (PMC4761790; doi:10.3389/fpls.2016.00194)
Supplement: Supplementary file 3 [file Table_3.DOCX]

Table S3. Additional plant functional traits. Means and standard deviation (sd) values for the four functional traits of most of the species tested by thermal analyses. Hmax, maximum plant height; SLA, specific leaf area; LDMC, leaf dry matter content

| **Species** | **Hmax (mm)** | | **SLA**  **(mm^2^ mg^–1^)** | | **LDMC**  **(mg g^–1^)** | | **Seed mass (μg)** | |
| --- | --- | --- | --- | --- | --- | --- | --- | --- |
|  | mean | sd | mean | sd | mean | sd | mean | sd |
| *Adenocarpus complicatus* | 789.4 | 113.9 | 10.2 | 3.7 | 313.2 | 40.3 | 4.4·10^4^ | 4703.2 |
| *Agrostis delicatula* | 20.5 | 6.8 | 11.4 | 2.0 | 397.3 | 44.5 | 63.7 | 13.8 |
| *Agrostis rupestris* | 37.3 | 9.4 | 15.9 | 1.1 | 376.9 | 16.9 | 52.7 | 18.3 |
| *Alchemilla saxatilis* | 49.8 | 21.1 | 13.4 | 2.0 | 371.9 | 28.9 | 384.4 | 116.6 |
| *Armeria caespitosa* | 16.2 | 4.3 | 15.1 | 2.2 | 350.7 | 34.9 | 641.7 | 94.0 |
| *Biscutella valentina* subsp. *pyrenaica* | 33.4 | 10.5 | 11.8 | 2.1 | 208.0 | 20.3 | 1202.9 | 231.7 |
| *Campanula herminii* | 19.9 | 5.6 | 24.2 | 2.5 | 214.9 | 17.6 | 60.4 | 5.1 |
| *Coincya monensis* subsp. *cheiranthos* | 37.2 | 13.3 | 8.3 | 8.8 | 163.1 | 23.0 | 1428.4 | 363.2 |
| *Cytisus oromediterraneus* | 502.5 | 120.8 | 14.3 | 2.2 | 258.2 | 32.1 | 1.1·10^4^ | 1526.1 |
| *Deschampsia flexuosa* | 27.6 | 9.4 | 7.7 | 1.2 | 396.9 | 30.7 | 617.1 | 130.0 |
| *Dianthus lusitanicus* | 33.6 | 10.9 | 8.9 | 1.3 | 273.6 | 18.0 | 1038.2 | 203.5 |
| *Erysimum penyalarense* | 30.6 | 11.4 | 20.1 | 4.3 | 201.4 | 33.8 | 1428.4 | 363.2 |
| *Festuca curvifolia* | 32.4 | 9.7 | 4.3 | 0.6 | 463.5 | 40.4 | 681.1 | 146.5 |
| *Gentiana lutea* | 182.0 | 60.5 | 8.9 | 1.0 | 267.3 | 14.7 | 964.7 | 243.6 |
| *Jasione crispa* | 15.0 | 4.5 | 18.4 | 3.7 | 241.6 | 27.6 | 49.6 | 5.2 |
| *Juniperus communis* subsp. *alpina* | 233.1 | 85.0 | 6.5 | 1.1 | 434.4 | 46.6 | 1.3·10^4^ | 1.2·10^4^ |
| *Jurinea humilis* | 7.0 | 3.3 | 10.1 | 1.5 | 266.3 | 34.9 | 7019.8 | 1239.3 |
| *Koeleria crassipes* | 25.9 | 3.8 | 12.6 | 0.6 | 390.7 | 11.0 | 236.7 | 37.1 |
| *Leucanthemopsis alpina* | 18.0 | 5.7 | 17.3 | 2.9 | 216.9 | 25.2 | 122.3 | 23.7 |
| *Linaria saxatilis* | 17.7 | 3.4 | 21.5 | 4.2 | 210.4 | 22.4 | 64.5 | 10.0 |
| *Luzula hispanica* | 44.4 | 9.7 | 13.3 | 2.6 | 305.8 | 30.2 | 253.6 | 34.1 |
| *Minuartia recurva* | 16.7 | 7.0 | 17.2 | 2.8 | 340.2 | 47.3 | 248.5 | 37.9 |
| *Murbeckiella boryi* | 19.2 | 14.5 | 24.2 | 5.1 | 153.4 | 26.8 | 121.2 | 12.8 |
| *Nardus stricta* | 85.3 | 15.6 | 6.8 | 0.8 | 401.7 | 17.2 | 239.0 | 29.1 |
| *Paronychia polygonifolia* | 20.0 | 7.9 | 18.4 | 3.2 | 315.1 | 44.2 | 351.5 | 44.7 |
| *Phyteuma hemisphaericum* | 22.2 | 6.6 | 18.1 | 2.3 | 269.4 | 17.4 | 73.0 | 12.4 |
| *Pilosella castellana* | 22.3 | 13.4 | 13.8 | 1.3 | 261.9 | 26.2 | 181.2 | 18.4 |
| *Pilosella vahlii* | 21.9 | 8.3 | 17.1 | 2.8 | 201.0 | 28.2 | 181.2 | 18.4 |
| *Rumex angiocarpus* | 43.8 | 19.6 | 10.6 | 1.5 | 189.3 | 24.4 | 335.6 | 65.1 |
| *Saxifraga pentadactylis* subsp. *Willkommiana* | 38.3 | 15.9 | 6.6 | 7.1 | 166.7 | 25.6 | 45.7 | 7.0 |
| *Sedum brevifolium* | 13.1 | 5.4 | 10.3 | 2.2 | 87.5 | 20.0 | 68.4 | 8.9 |
| *Sedum candollei* | 12.7 | 3.0 | 19.3 | 2.7 | 87.8 | 9.8 | 21.6 | 2.4 |
| *Senecio carpetanus* | 313.8 | 89.6 | 10.3 | 1.5 | 189.6 | 29.3 | 2455.6 | 357.4 |
| *Silene boryi* | 34.4 | 7.2 | 14.3 | 2.4 | 244.5 | 24.4 | 778.3 | 192.7 |
| *Silene ciliata* | 19.6 | 5.1 | 14.1 | 1.9 | 207.0 | 24.2 | 348.9 | 54.0 |
| *Solidago virgaurea* subsp. *fallit-tirones* | 23.5 | 6.0 | 12.3 | 1.0 | 286.1 | 16.5 | 862.0 | 159.4 |
| *Thymus praecox* subsp. *penyalarensis* | 21.1 | 4.8 | 14.9 | 1.7 | 320.0 | 27.4 | 197.5 | 37.5 |
| *Veronica fruticans* subsp. *cantabrica* | 17.1 | 5.2 | 12.9 | 1.8 | 308.7 | 29.6 | 157.1 | 21.7 |
